# Editorial: Glial cells and immune cells in neuroinflammatory and neurodegenerative diseases

**DOI:** 10.3389/fnagi.2022.1120649

**Published:** 2023-01-05

**Authors:** Rui Wang, Haigang Ren, Li-Fang Hu, Ning Song, Ling Long, Zili You, Guanghui Wang

**Affiliations:** ^1^Center of Translational Medicine, The First People's Hospital of Taicang, Taicang Affiliated Hospital of Soochow University, Suzhou, Jiangsu, China; ^2^Laboratory of Molecular Neuropathology, Jiangsu Key Laboratory of Neuropsychiatric Diseases and College of Pharmaceutical Sciences, Soochow University, Suzhou, Jiangsu, China; ^3^Institute of Neuroscience, Soochow University, Suzhou, Jiangsu, China; ^4^School of Basic Medicine, Qingdao University, Qingdao, Shandong, China; ^5^Department of Neurology, Third Affiliated Hospital of Sun Yat-sen University, Guangzhou, Guangdong, China; ^6^School of Life Science and Technology, University of Electronic Science and Technology of China, Chengdu, Sichuan, China

**Keywords:** neuroinflammation, microglia, astrocyte, neurodegeneration, heterogeneity, glymphatic system

Immune dysfunction and inflammation are involved in autoimmune neurological and neurodegenerative disorders (NDs) (Tansey et al., 2022). It is now clear that the dysfunction of glial cells, including microglia, astrocytes and oligodendrocytes, contributes to neuroinflammation in disease pathogenesis. Genome-wide association studies have identified some disease-associated gene mutations that are highly expressed in glial cells, which lead to autonomous disturbances of glial cells and may initiate diseases or induce neuroinflammation to contribute to disease pathogenesis (Shi and Holtzman, 2018). Recently, emerging novel genomic technologies have enabled the characterization of distinct types of glial cells in response to distinct disease conditions (Colonna and Brioschi, 2020). The temporal and spatial heterogeneity of glial cells has either beneficial or detrimental effects on disease progression. Moreover, bidirectional communication between glial cells in the central nervous system (CNS) and other immune cells in the peripheral tissues is essential for brain homeostasis. Disorders of the peripheral immune cells also contribute to the reactivity of glial cells in the CNS (Tansey et al., 2022). The manuscripts in this Research Topic focus on the mechanism underlying how glial cells contribute to the pathogenesis of neurodegeneration and other neuroinflammatory disorders. We highlight two specific themes in this topic: (1) the roles of neuroinflammation caused by glial cell dysfunction in disease pathogenesis and (2) the heterogeneity of glial cells in response to disease-associated conditions.

In autoimmune disorders and NDs, microglia-associated neuroinflammation plays a crucial role in disease pathogenesis. Reactive microglia in a chronic state secrete multiple cytokines and inflammatory mediators into the surrounding microenvironment, leading to the activation of other glial cells and damage to neurons (Shi and Holtzman, 2018). Mild cognitive impairment, as a key risk factor for Alzheimer's disease and Parkinson's disease (PD)-associated dementia, has been reported to be associated with neuroinflammation (Liu et al., 2021). Cai et al. systematically analyzed the literature in association with neuroinflammation-induced mild cognitive impairment in recent years using bibliometric analysis, showing that the activation of microglia through receptor triggering receptor expressed on myeloid cells 2 (TREM2) and ligand apolipoprotein E4 (ApoE4) has a role in the regulation of microglia. Liu et al. comprehensively reviewed the roles of mitophagy in the maintenance of microglial functions and the regulation of neuroinflammation in neurological disorders. Mitophagy contributes to mitochondrial homeostasis and clearance of damaged mitochondria, which reduce oxidative stress and restore ATP levels. The authors summarized the mechanisms of mitophagy regulation and the influence of the pathologic factors on mitophagy. In addition, they comprehensively summarized the role of microglial mitophagy in different NDs and aging. Furthermore, they described the regulation of mitophagy by multiple factors that modify microglial activity. Russo and Riessland discussed the relationships between cell senescence and inflammatory activation in PD. They summarized the evidence of cell senescence in neurons and different glial cells in the brain, as well as T cells in the periphery in PD. They discussed that senescence-associated secretory phenotype activates local glial cells as well as peripheral immune cells, which upregulates the expressions of inflammatory factors in blood and brain in PD patients, leading to the damage of dopaminergic neurons in the substantia nigra. In addition to NDs, Zhen-Gang et al. discussed crosstalk among glial cells or glial cells and peripheral immune cells that plays roles in spinal cord injury. Moreover, peripheral organs, including the spleen and gut, are involved in SCI under different conditions and promote or protect against spinal cord injury.

Heterogeneity in microglial phenotypes has received much attention in recent years due to the development of single-cell sequencing (Colonna and Brioschi, 2020). As immune cells in the CNS, microglia are sensitive to changes in the microenvironment in the brain. An important feature of the heterogeneous phenotype of microglia is regional specificity in gene expression. Brandi et al. examined microglial heterogeneity in response to systemic inflammation and found brain region-specific activation of microglia and astrocytes. Moreover, they showed that CX3CR1 contributes to microglial activation after systemic lipopolysaccharide administration. Their study provided a novel mechanism, explaining that the brain region-specific heterogeneity of microglia may contribute to the susceptibility of midbrain dopaminergic neurons in PD.

The CNS was previously thought to lack the lymphatic vessels that are involved in the removal of metabolic waste products from the brain. Recent studies have shown that there is a specific type of lymphatic system called the “glymphatic system” that is composed of a glial-dependent perivascular network to support the drainage of waste in the brain (Plog and Nedergaard, 2018). Zhang et al. reviewed the mechanisms underlying the regulation of the glymphatic system and the roles of the glymphatic pathway in neurological diseases. In addition, they discussed the relationship between sleep and the glymphatic system, emphasizing that impairment of the glymphatic system by pathological factors in glial cells may be an early event in sleep and mental disorders.

Taken together, this Research Topic summarizes the mechanisms underlying glial cell dysfunction that contribute to neuroinflammation and the pathogenesis of NDs.

## Author contributions

RW drafted the manuscript. HR, L-FH, NS, LL, and ZY provided suggestions. GW revised and finalized the manuscript. All authors contributed to the article and approved the submitted version.
